# Can the adenosine triphosphate (ATP) bioluminescence assay be used as an indicator for hospital cleaning? – A pilot study

**DOI:** 10.3205/dgkh000462

**Published:** 2024-02-21

**Authors:** Valerie Niephaus, Nina Parohl, Sabine Heiligtag, Henning Reuter, Reiner Hackler, Walter Popp

**Affiliations:** 1 Evang. Kliniken Essen-Mitte, Essen, Germany; 2 HyKoMed GmbH, Dortmund, Germany; 3 3M Deutschland GmbH, Neuss, Germany

**Keywords:** ATP, adenosine triphosphate bioluminescence, hospital cleaning, cleaning monitoring, healthcare-associated infections

## Abstract

**Background:**

In hospital cleaning, there is currently no standard for uniform monitoring of surface cleaning, either in Germany or internationally. One possibility for monitoring is the use of so-called objective methods for checking cleaning performance (e.g. fluorescence or adenosine triphosphate (ATP) method).

**Aim:**

The aim of the study was to monitor and evaluate the implementation of the adenosine triphosphate (ATP) bioluminescence assay as a cleaning indicator in everyday hospital cleaning, in order to verify its utility and effectiveness.

**Methods:**

In three phases, five frequently touched surfaces were examined with the ATP bioluminescence assay at different time points. 846 measurements were performed on the dermatology ward of a university hospital (phase 1), 1,350 measurements were performed on five different wards of the university hospital (phase 2), and 1,044 measurements were performed on five wards of another large hospital (phase 3). For this purpose, one structurally old and one structurally new ward as well as an intensive care unit (ICU), an outpatient clinic and a radiology department were selected for phases 2 and 3.

**Results:**

With the ATP bioluminescence method, we were able to demonstrate a reduction in values after cleaning: before cleaning mean of ATP, 907 relative light units (RLU) (95% confidence interval [CI] 777; 1,038); after cleaning mean=286 RLU (CI=233; 495) (phase 1) and by intervention (five hours after daily cleaning mean=360 RLU (CI=303; 428); five hours after daily cleaning and two additional cleanings mean=128 RLU (CI=107; 152) (phase 3). The ATP values increased five hours after cleaning in phases 1 and 2, and eight hours after cleaning in phase 3. The structurally old wards had the highest ATP content, the ICU and the radiology department, among others, the lowest. In all phases, door handles showed both a reduction after cleaning or intervention and a subsequent increase in ATP values. Chair armrests, examination tables and door handles had high ATP values overall.

**Conclusion:**

The study shows ward differences both for cleaning effects and for the soiling characteristics of surfaces during the course of the day. In addition, it demonstrates the benefit of intermediate cleaning twice a day. It is noteworthy that structurally old stations and older inventory were more heavily soiled and, in some cases, more difficult to clean.

The results show that the ATP bioluminescence method is suitable for detecting cleaning effects and can be used in everyday clinical practice for simple cleaning monitoring. Furthermore, it enables the detection of risk surfaces and easy-to-clean surfaces with significant re-soiling.

## Zusammenfassung

**Hintergrund:**

In der Krankenhausreinigung gibt es weder in Deutschland noch international derzeit einen Standard für ein einheitliches Monitoring der Flächenreinigung. Eine Möglichkeit der objektiven Kontrolle ist der Einsatz sog. objektiver Methoden zur Überprüfung der Reinigungsleistung, z.B. mit der Fluoreszenz- oder Adenosintriphosphat (ATP)-Methode.

**Zielsetzung:**

Ziel der Studie war die Implementierung der ATP-Biolumineszenz-Methode als Reinigungsindikator im Krankenhausalltag und die Bewertung deren Aussagekraft.

**Methoden:**

In 3 Phasen wurden 5 häufig angefasste Oberflächen zu verschiedenen Zeitpunkten untersucht. In Phase 1 wurden 846 Messungen auf der dermatologischen Station einer Universitätsklinik, in Phase 2 1.350 Messungen auf fünf verschiedenen Stationen der Universitätsklinik und in Phase 3 1.044 Messungen auf 5 Stationen eines anderen großen Krankenhauses durchgeführt. Für Phase 2 und 3 wurden jeweils eine baulich alte und neue Station sowie eine Intensivstation, Ambulanz und Röntgenabteilung ausgewählt.

**Ergebnisse:**

Die ATP-Biolumineszenz-Methode konnte eine Reduktion der Werte nach der Reinigung (vor der Reinigung ATP-Mittelwert (M) 907 RLU; 95% Konfidenzintervall (CI) 777; 1.038, nach der Reinigung M=286 RLU; CI=233; 495) (Phase 1) nachweisen sowie durch die Intervention 5 h nach der täglichen Reinigung (M=360 RLU (CI=303; 428); 5 h nach täglicher Reinigung und zweimaliger zusätzlicher Reinigung M=128 RLU, CI=107; 152) (Phase 3). Die ATP-Werte stiegen 5 h (Phase 1 und 2) bzw. 8 h nach der Reinigung wieder an (Phase 3). Die baulich alten Stationen wiesen den höchsten ATP-Gehalt auf, die Intensivstationen und die Röntgenabteilungen u.a. den niedrigsten. Die Türklinke wies in allen Phasen sowohl eine Reduktion nach Reinigung bzw. Intervention als auch einen nachfolgenden Anstieg der ATP-Werte auf. Die Stuhlarmlehne, die Ambulanzliege und der Türgriff wiesen insgesamt hohe ATP-Werte auf.

**Fazit:**

Die Studie kann Stationsunterschiede sowohl für Reinigungseffekte als auch für die Verschmutzung von Oberflächen im Tagesverlauf aufzeigen. Darüber hinaus weist sie den positiven Nutzen einer zweimaligen Zwischenreinigung nach. Auffallend ist, dass baulich alte Stationen und älteres Inventar stärker verschmutzen und zum Teil schwieriger zu reinigen sind.

Die Ergebnisse zeigen, dass die ATP-Biolumineszenz-Methode zum Nachweis von Reinigungseffekten geeignet ist und im Klinikalltag zur einfachen Reinigungskontrolle eingesetzt werden kann. Darüber hinaus ermöglicht sie die Bestimmung von Risikoflächen bzw. leicht zu reinigenden Flächen mit erheblicher Wiederverschmutzung.

## Introduction

Nosocomial infections with multidrug-resistant pathogens (MDRO) are a major problem for many hospitals, due to the more complicated therapy, the increased length of stay and high costs for the healthcare system [1]. Environmental cleaning, especially of surfaces close to patients, is of great importance for the prevention of nosocomial infections [2], [3]. In addition to direct personal contact, contaminated objects are also vectors for pathogens [4]. There are already many interventions to reduce nosocomial infections, such as hand antisepsis campaigns and standardised procedures [5], [6], [7].

However, surface disinfection is still given too little importance in the daily routine of many hospitals, although recent studies show that hospital-acquired infections can result from contaminated surfaces, especially close to patients [8], [9], [10]. Therefore, it is necessary to implement objective methods for cleaning monitoring in hospitals. One of these is the adenosine triphosphate (ATP) bioluminescence assay, which has been established in hygiene research for many years. It should be mentioned that guidelines and recommendations for cleaning monitoring have been published in the meantime. For this purpose, DIN 13063 and the Commission for Hospital Hygiene and Vaccination Prevention (KRINKO for short) specify the use of objective methods [10], [11]. Based on this, the present study reviews the applicability of the ATP bioluminescence method as cleaning monitoring in daily hospital practice.

## Method

In 3 phases, frequently touched surfaces were examined by means of ATP measurements from January 2015 to August 2016 in two different hospitals in a German city. Prior to this, these surfaces were selected by inspection of the wards, provided they had been cleaned by the cleaning staff. Different disinfectants were used for surface disinfection, such as Perform^®^ 0.5% (Schülke & Mayr GmbH, active agent: active oxygen, spectrum of activity: bactericidal, levurocidal, fungicidal, virucidal, sporicidal), Incidin™ Plus 0.5% (Ecolab Deutschland GmbH, active agent: Glucoprotamin™, spectrum of activity: bactericidal, levurocidal, virucidal, tuberculocidal, mycobactericidal) (phase 1 and 2) and Terralin^®^ Protect 0.5% (Schülke & Mayr GmbH, active agent: quaternary ammonium compounds; spectrum of activity: bactericidal, levurocidal, tuberculocidal and virucidal at low load) (phase 3). The exposure time may be varied for the respective cleaning effect. The disinfectants have shown an influence on the results of the ATP measurements [12], [13], [14]. Sampling was done only after the surface was completely dry to eliminate the influence of disinfectant residues.

The cleaning staff was informed about the study. Each sampled room was occupied by at least one patient. The aim was to determine the cleaning effect and factors influencing soiling development during the course of the day by ATP measurement.

### Phase 1

On each of four days, six frequently touched surfaces were examined in 10 rooms of the dermatology ward of a university hospital. Samples were taken before, immediately after and five hours after daily cleaning. A total of 846 measurements were taken by two samplers.

### Phase 2

The focus was on surface measurements from five wards with different requirements. On five days, on five frequently touched surfaces, a total of 1,350 measurements were taken by a sampler 15 minutes after daily cleaning and five hours afterwards. A structurally old ward (urology), a structurally new ward (trauma surgery), an outpatient clinic (trauma surgery outpatient clinic), a radiology department and an intensive care unit were selected. The order of the surface measurements per room was randomised.

### Phase 3

The focus was to prove the effect of an intervention and the applicability of the ATP bioluminescence method on five wards in another large hospital in Essen. For this purpose, a total of 1,044 measurements were taken weekly by one sampler on a control day and an intervention day. On intervention days, an additional intermediate cleaning of the surfaces took place 1.5 hours and three hours after the daily cleaning. 

There was always a rest day between the control day and the intervention day. Therefore, the surfaces were examined immediately after cleaning and five and eight hours afterwards. 

Two rooms each in the oncology ward as a structurally old ward, the private ward as a structurally new ward, and the multidisciplinary emergency unit as an outpatient department, were studied in three weeks. The intensive care unit with three rooms was also examined for three weeks. The radiology’s only X-ray room was assessed for six weeks.

### Samplings

For sampling, 3M™ Clean-Trace™ ATP surface tests and the 3M™ Clean-Trace™ NGi luminometer were used according to the manufacturer’s instructions. The samplers also received training from a 3M™ Deutschland GmbH employee on the proper use of the surface tests and the luminometer. Templates of 4x4 cm and 8x2 cm were used for sampling to obtain a standardised area of 16 cm².

### Statistical analysis

Statistical analysis was performed using Minitab 17 (Minitab Inc., State College, Pennsylvania, USA). Descriptive statistics were presented using box-plot and interval plots. Inductive statistics were performed using generalised linear mixed models (GLM) to determine significant differences between means of ATP content (dependent variable) for fixed factors (e.g., time, surface, ward, room, intervention). p<0.1 was considered statistically significant, as determined by the overall F-test and t-distribution. The raw data were not normally distributed, so they were transformed using log-normal distribution. The analysis was based on replacing outliers that were three standard deviations away from the expected value µ, calculated by the model. Only significant terms were used for the model [15].

## Results

### Phase 1

**Figure 1:**
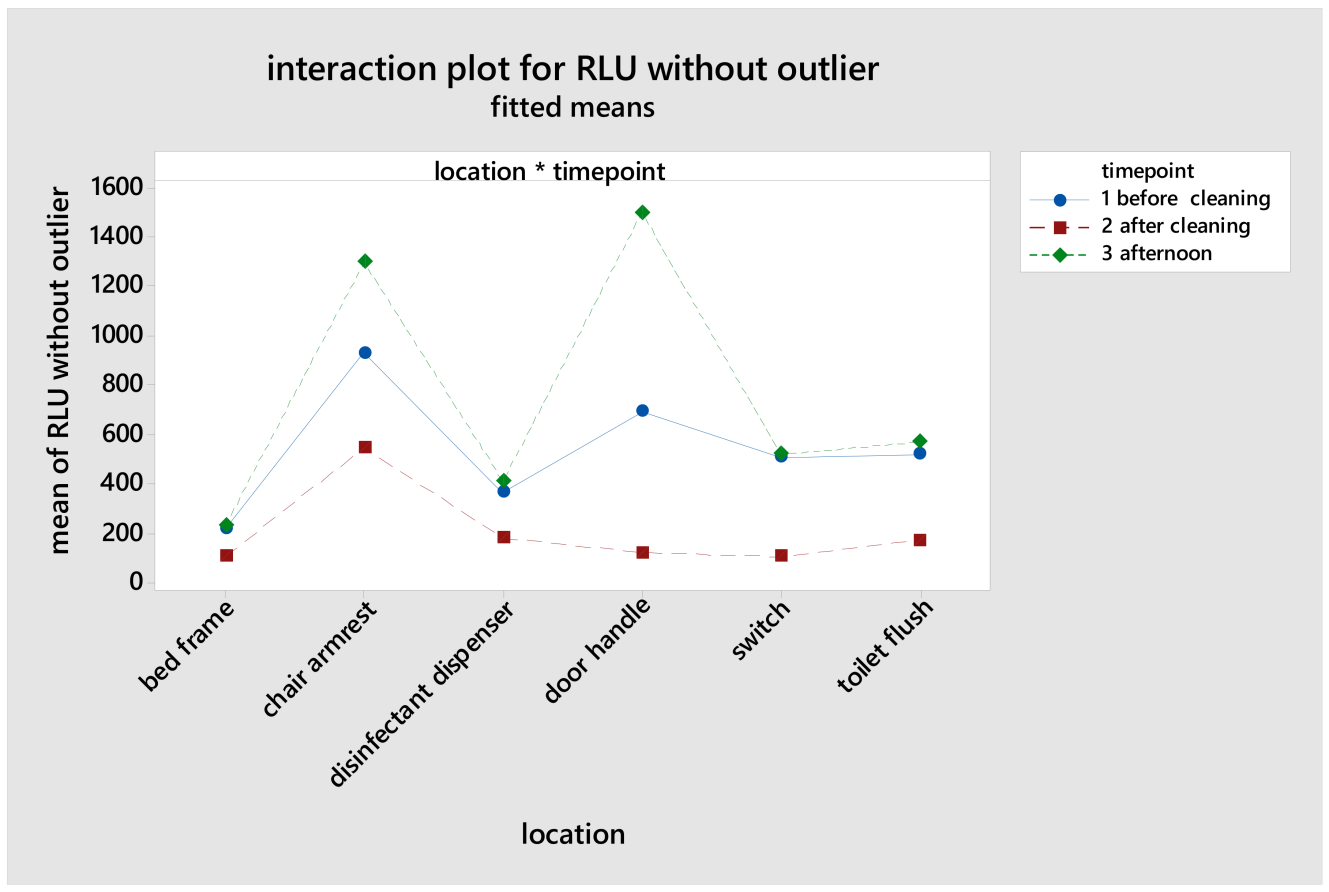
Interaction plot for relative light units (RLU), timepoint and location, phase 1.

The factor “time” had the greatest influence on the ATP content, with a decrease after cleaning and an increase five hours afterwards (afternoon) (Table 1 (Tab. 1)). Table 2 (Tab. 2) and Figure 1 (Fig. 1) give an overview of ATP values for the point of time and surfaces. Chair armrests had the highest average ATP content. The greatest reduction in ATP values was found for door handles. Five hours after cleaning, the greatest increase over the course of the day was also found for door handles; these changes were significantly different. There was a significant difference between the investigators. This effect was only significant for the time before cleaning, but not after cleaning. The analysis showed no significant difference between the cleaners. The factor “room number” showed a large range of mean values. 

### Phase 2

**Figure 2:**
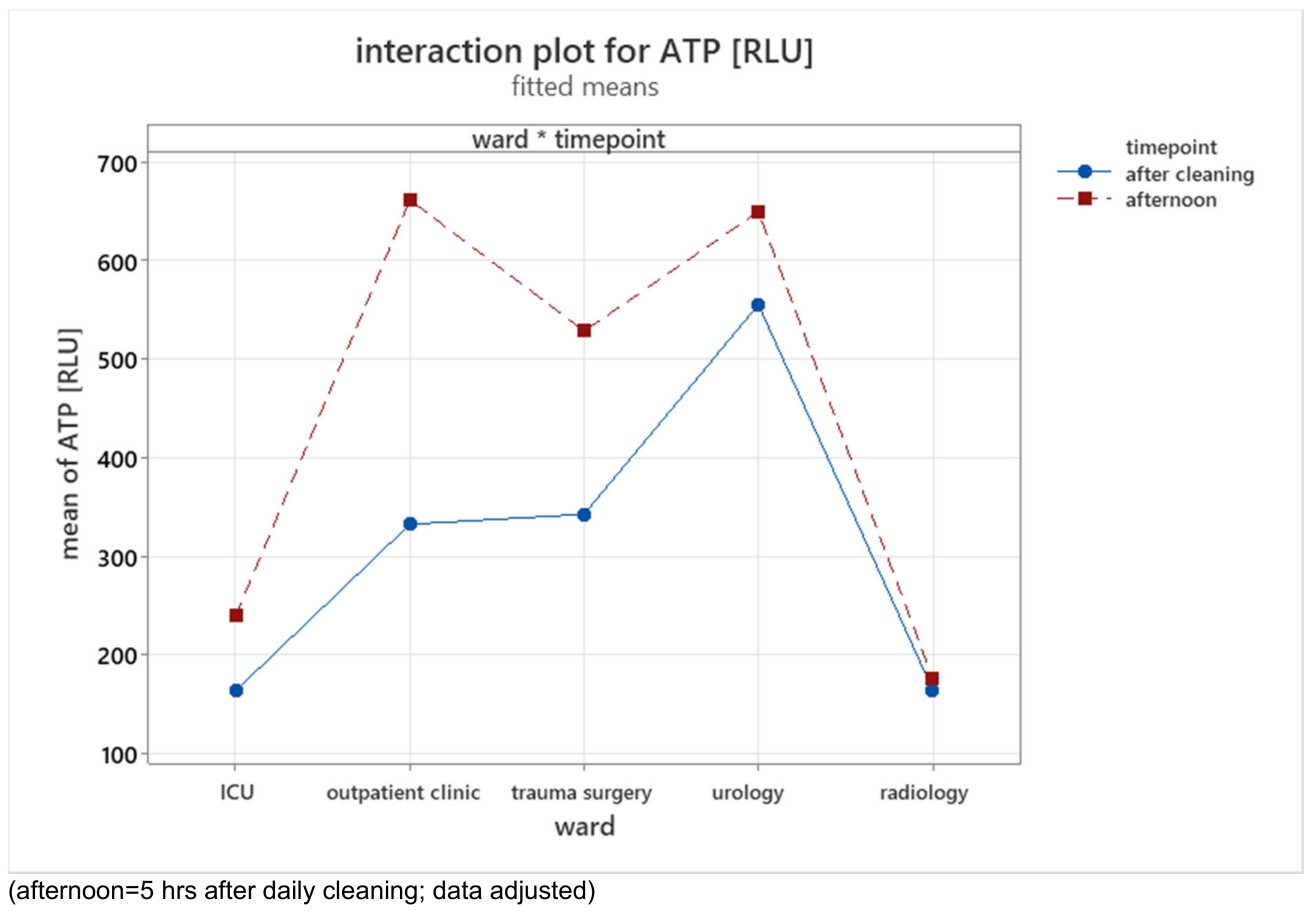
Interaction plot for means of ATP in relative light units (RLU), phase 2.

The ATP content increased five hours after cleaning. There were clear ward differences. The structurally old urology ward had the highest ATP content. The outpatient department and the structurally new trauma surgery ward had a significantly lower ATP content. The lowest values were found for the ICU and radiology department (Figure 2 (Fig. 2)). 

For all wards, an increase in values was found five hours after cleaning. This increase varied and was highest for the outpatient department and lowest for the radiology department (Table 3 (Tab. 3)).

In phase 2, there were surfaces with high ATP values, such as door handles, chair armrests (trauma surgery and urology ward) and examination tables (outpatient clinic). Overall, door handles also exhibited a high increase in values five hours after cleaning. 

### Phase 3

**Figure 3:**
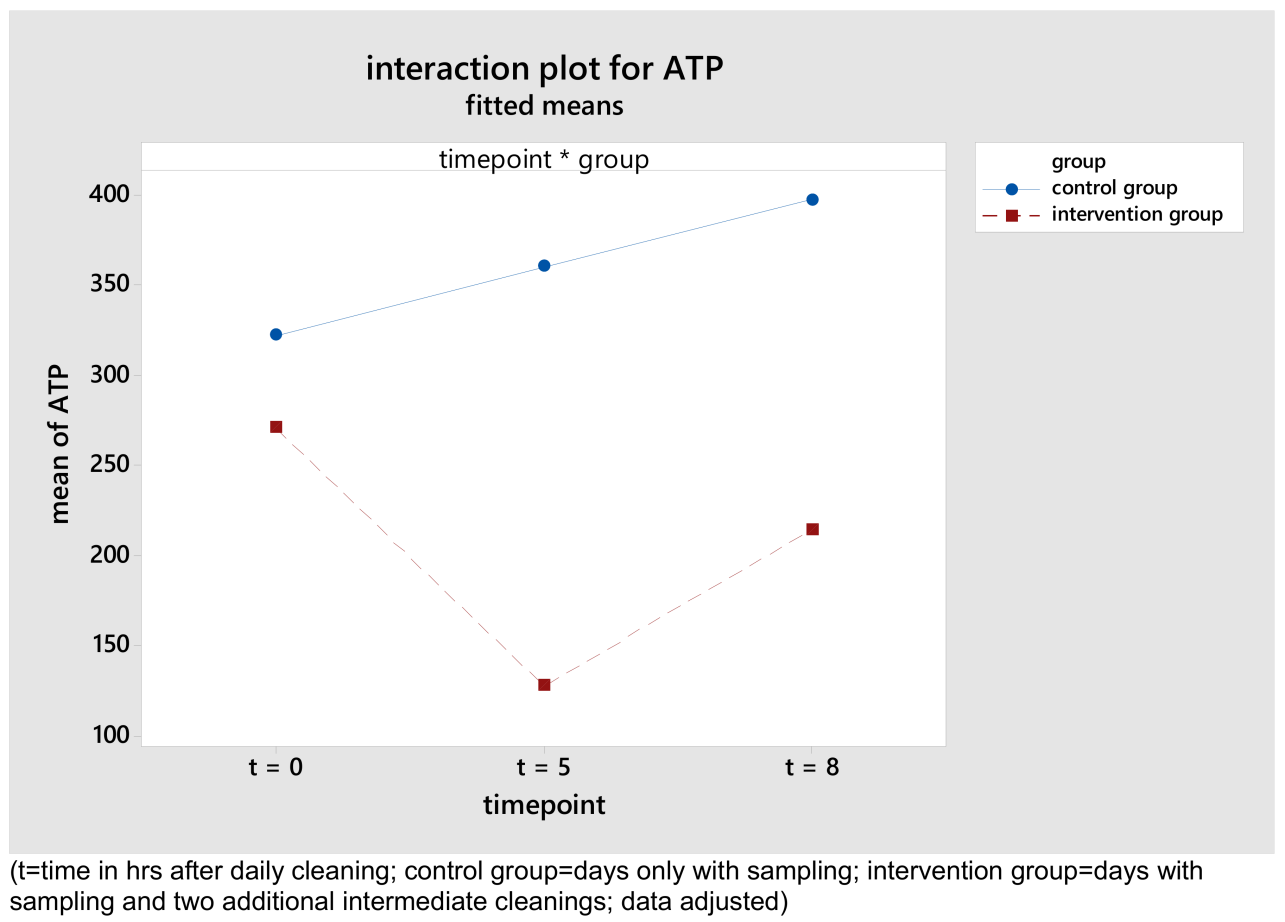
Interaction diagrams for mean of ATP in relative light units (RLU), phase 3.

The intervention group had a significantly reduced ATP content compared to the control group (Table 4 (Tab. 4)). On control days, the values increased continuously, in contrast to the values on intervention days. There, a significant reduction of the ATP content with a subsequent increase observed after the intervention. Figure 3 (Fig. 3) illustrates the difference between control and intervention group for the point of time.

In the intervention group, the ATP values of the wards converged, and the value differences decreased. Compared to the control group, a reduction of the standard deviations and standard errors was noticeable in the intervention group. 

The structurally old oncology ward and the outpatient clinic had significantly higher ATP values than the other wards. In particular, chair surfaces and table tops (oncology) as well as examination couch (outpatient department) had high ATP values. For door handles, the ATP content increased most significantly after the intervention, up to 8 hours after daily cleaning.

## Discussion

Like other studies, we were able to demonstrate the positive effect of both daily cleaning [16], [17], [18], [19] and an intervention [20], [21], [22] on surface contamination using the ATP bioluminescence method. An interesting aspect is offered by Smith et al. [23], who were able to demonstrate a post-intervention effect even after a washout phase. Their results show not only the short-term, but also and especially the long-term effect of an intervention program. In our study, there was no longer-term follow-up. However, it does show the lasting effect of the intervention over the course of the day. Furthermore, as in our case, there is a reduction in variability due to the intervention [24]. Thus, the method may serve to improve and monitor existing cleaning practices in terms of reducing nosocomial infections as a continuing health risk [25].

Moreover, we were able to identify risk surfaces with different cleaning requirements. On the one hand, door handles, cupboard handles and chair surfaces were easy to clean. Other authors also describe an ATP reduction for door handles after cleaning [3] or through an intervention (3 periods, cleaning monitoring by fluorescence markers, additional UV cleaning and enhanced cleaning after the use of ATP bioluminescence measurements) [26]. In addition, we found a significant increase in contamination over the course of the day for door handles in all three phases. An unrealistic option is more frequent intermediate cleaning of such surfaces. If the 5 WHO moments of hand antisepsis are followed, these potential infection routes for staff can be interrupted. On the other hand, there were surfaces with more difficult cleaning characteristics and overall high contamination. Other authors also documented such surfaces [3], [17], [19], [27]. These can have a considerable influence on the ward level. For the oncology chair surface, there is hardly any effect of the intervention, despite correctly performed cleaning. This may be due to potentially porous, scratched and long-stressed surface materials [28]. For surfaces with structurally demanding cleaning characteristics, the choice of disinfectant and detergent [29], cleaning cloth and application of mechanical force is important [22], [30]. However, one should not draw conclusions from one surface contamination to the contamination of other surfaces of a whole room or ward [21]. In particular, heavily contaminated surfaces should be investigated as to their nature and cleaning method. To ensure the cleaning or disinfecting cleaning of surfaces, the Commission for Hospital Hygiene and Infection Prevention (KRINKO) at the Robert Koch Institute Berlin recommends that hygienically relevant surfaces are to be cleaned and disinfected safely and that no material damage be caused by the cleaning or disinfecting surface cleaning. Before purchasing new items, e.g., floor coverings and furniture, it is recommended that the manufacturer confirm the material compatibility regarding disinfectability [10]. The KRINKO and its voluntary work are legitimised by the mandate according to § 23 of the German Infection Protection Act to establish binding principles and standards for prevention measures. Adjustments in cleaning procedures, especially mechanics and duration, may need to be made and surface material may need to be replaced. It is also important to sensitize cleaning, nursing and medical staff to the cleaning of such surfaces.

In phases 2 and 3, the structurally old wards (urology ward, oncology ward) with the comparatively oldest inventory had the highest ATP values. The intensive care units and radiology departments, on the other hand, had the lowest ATP values. Possible reasons could be a fixed room allocation, greater motivation [21] of the nursing staff, as well as more frequent intermediate cleaning. Other authors also found lower ATP values for intensive care units [22], [31]. In our study, there were clear differences between the wards during the course of the day, which also have different levels of contamination due to full occupancy and increased patient turnover, among other factors [6]. For example, the outpatient department in phase 2 showed the greatest increase in contamination, while ATP values in phase 3 decreased on control days. It should be noted that due to different work structures and differing ward requirements, a direct comparison between wards, hospitals, countries and even surfaces is difficult [32]. Furthermore, we have not determined any benchmark for the purity classification, as no generally applicable benchmark has been found so far [27], [33], [34]. Therefore, like other authors, we recommend determining the internal limits for each hospital individually [22], [35].

We found that at least before cleaning, the sampling persons showed a significant difference (phase 1). Therefore, we conducted the further measurements with only one sampler, as Knape et al. [21] did, to reduce the systematic error. Moreover, we found that all cleaners had similar cleaning efficiencies. This indicates a standardised cleaning protocol. Its application and adaptation contribute significantly to the improvement of surface cleaning [36], [37]. Another reason is the Hawthorne effect, as all cleaners were informed about the study in advance.

We did not conduct microbiological examinations of the surfaces, as many previous studies have shown little or no correlation with the ATP bioluminescence assay [17], [18], [19], [38], [39]. It should be noted that the determination of ATP only quantitatively measures organic material without any differentiation, even between viable and non-viable organisms [40]. It can be concluded that the measurement of ATP can verify the effectiveness of purification, but not determine the prevailing microbial load.

In addition to many non-detectable influencing factors on surface purity in the hospital, however, there are also various influencing factors as a limitation of the ATP bioluminescence method. Among other things, this results in high variability, which we as well as other authors documented [19], [27], [28], [37], [41]. The choice of disinfectant can also influence the ATP results [12], [25], [32]. Therefore, one should not use different disinfectants in comparisons [13]. However, in addition to detergent and disinfectant residues, ATP measurements can also be influenced by worn surfaces, softeners, microfiber cloths and ammonium compounds in detergents [42]. Considering various factors, comparability between studies and hospitals is difficult, also due to significant differences in study designs, diverse hospital structures and requirements [25].

## Conclusion

Our results allow the evaluation of the ATP bioluminescence method for routine monitoring of cleaning in different hospitals. It is fast, cost-effective and useful for assessing interventions, but high readings do not indicate a risk of infection for patients [42]. ATP measurements offer a major advantage in their ease of use and they can provide quick feedback through self-application, highlight potential cleaning deficits and thus increase cleaning performance and staff motivation. In addition, risk surfaces are highlighted and can be examined more closely for their condition and cleaning requirements. However, before implementing the ATP bioluminescence assay, we recommend that each hospital establish its own reference values to determine individual contamination levels and associated benchmarks. 

In summary, our study confirms the use of the ATP bioluminescence method for cleaning assessment in hospitals, in accordance with current studies [34], [35]. The efficiency of a cleaning process, surface-specific effects and the evaluation of existing cleaning protocols can be shown by ATP measurements and allow direct interpretation considering various influencing factors.

According to Ferreira et al. [43], the fluorescence method and the optical control are well suited for monitoring compliance with the cleaning specifications, while methods that check the microbiological load give a better indication of an actual risk of infection and the effectiveness of the disinfection. Irrespective of the use of a measuring method, the observation of process sequences by means of a checklist is necessary to assess the quality of disinfecting surface cleaning. The repeat intervals must be set according to the results of the audit. If monitoring is used specifically to improve quality, the choice of method is secondary [10].

## Notes

### Author’s ORCID

- Valerie Niephaus: 0009-0005-5526-2539

### Competing interests

The study was financially supported by 3M Germany, Neuss, Germany. Three authors are employed by 3M Germany, Neuss, Germany.

## Figures and Tables

**Table 1:**
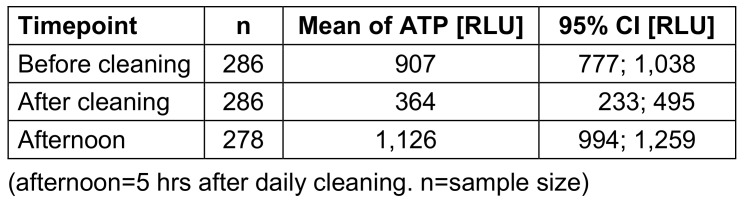
Mean value of relative light units (RLU) with 95%-confidence interval (CI) of timepoint, phase 1.

**Table 2:**
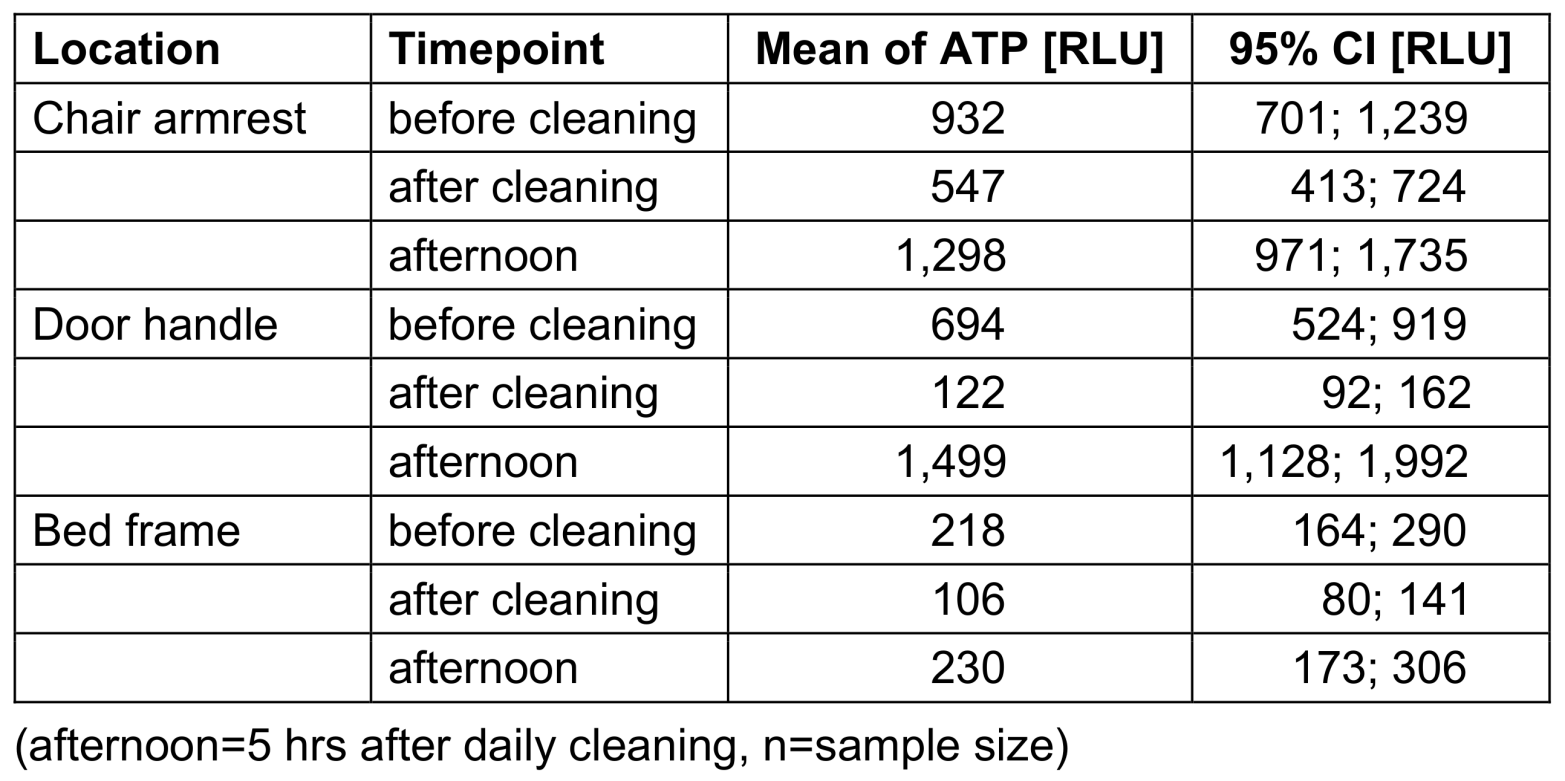
Mean value of relative light units (RLU) with 95%-confidence interval (CI) of location with highest and lowest contamination.

**Table 3:**
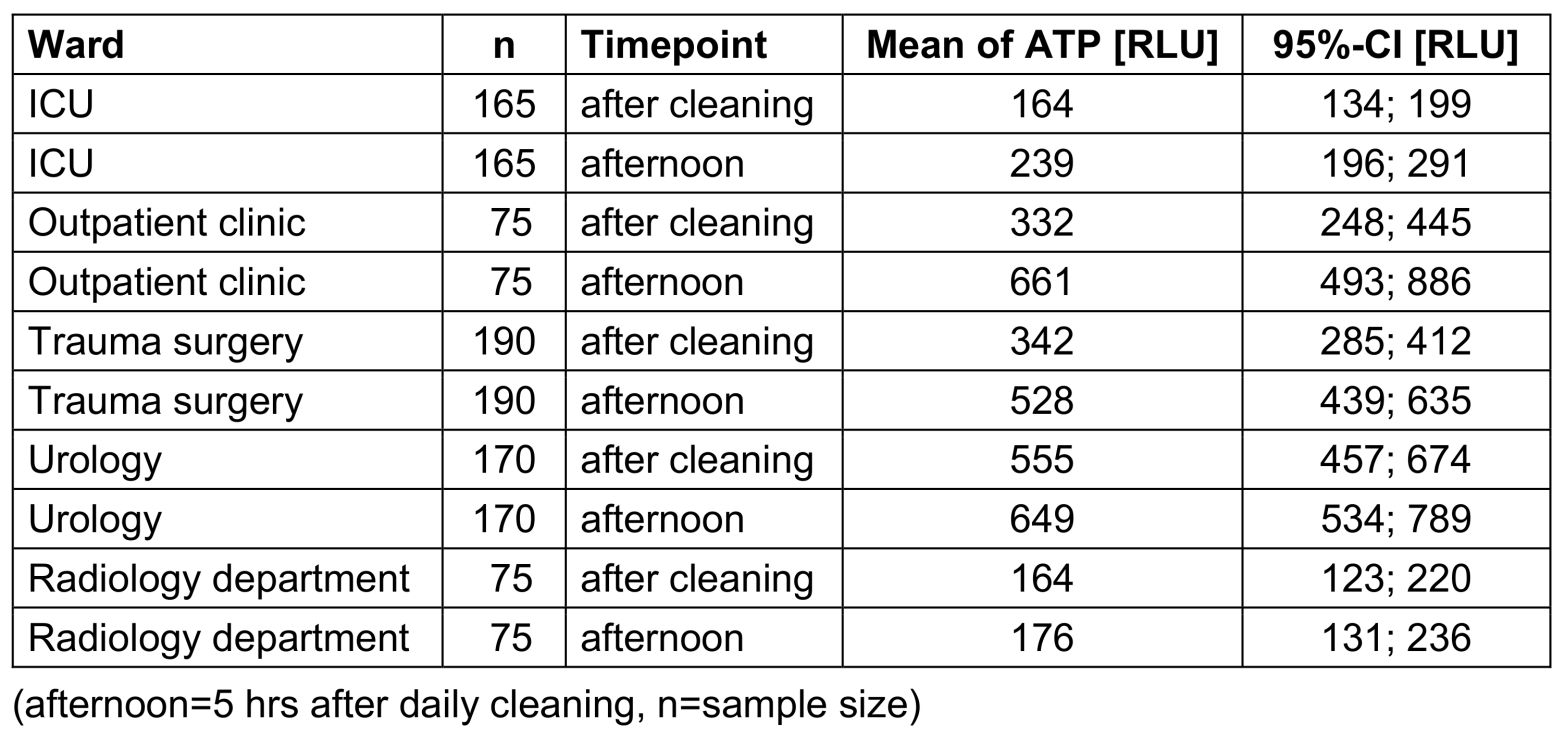
Mean values and 95%-confidence interval (CI) of relative light units (RLU) in different wards.

**Table 4:**
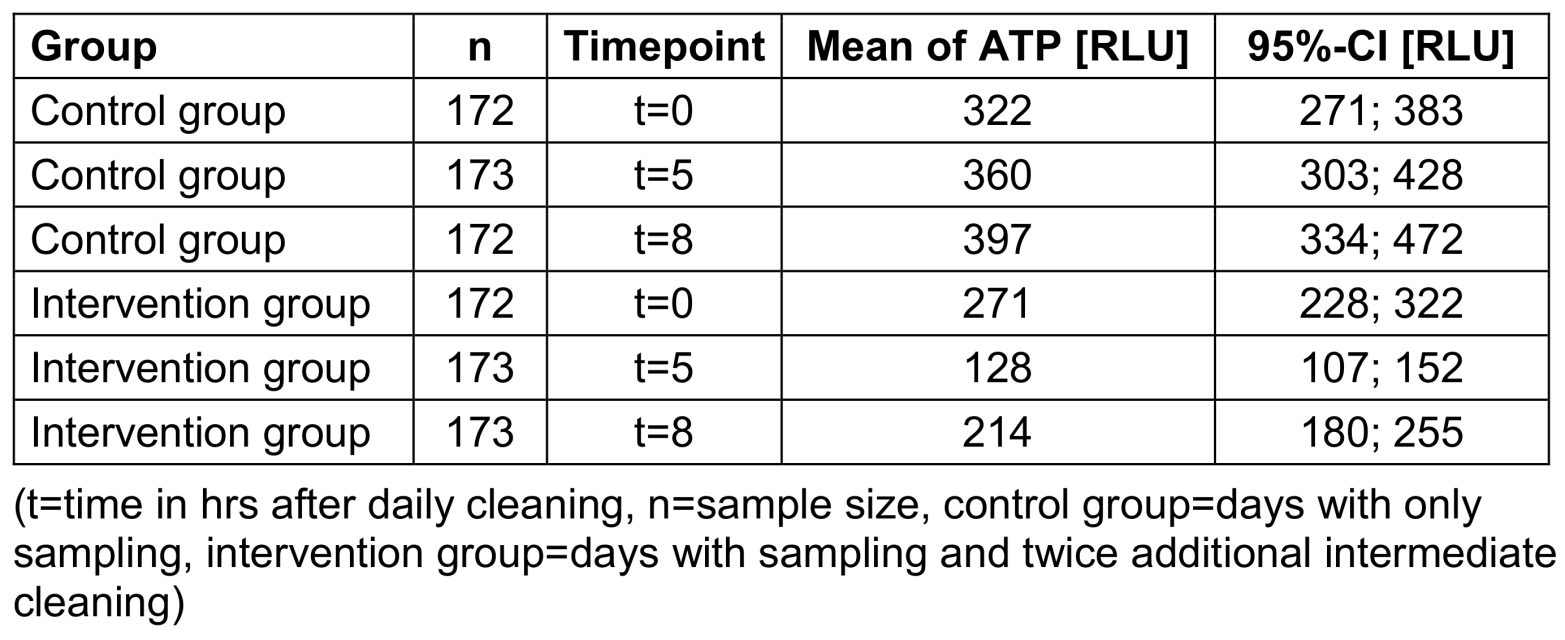
Mean values and 95%-confidence interval (CI) of ATP in relative light units (RLU) for group and timepoint.
